# EXAMINING OPPORTUNITIES TO ALIGN WITH THE UNIFYING GOAL OF HELPING CAREGIVERS: A STRENGTH-BASED MODEL

**DOI:** 10.1093/geroni/igad104.1226

**Published:** 2023-12-21

**Authors:** Jeong Eun Lee, Naomi Meinertz, Tina Savla

## Abstract

Caregiving is a complex and multifaceted experience, yet it is often addressed from a deficit model. Recent research indicates that strength-based interventions or programs from person- centered approach demonstrate improvements in caregivers’ experiences. The purpose of this symposium are is to highlight a collection of studies that each bring a unique perspective to family the care issues by, reporting improved caregiver psychosocial factors contributing to caregiver outcomes and strength or person centered approach to enhance caregiver outcomes. The first session will demonstrate the effectiveness of a person-centered caregiver intervention, Powerful Tools for Caregivers (PTC), in reducing depressive symptoms, improving self-efficacy, and utilizing emotion management skills. The second session will share a baseline data from a live online intervention, Chronic Grief Management Intervention-Video (CGMI-V), and identifying intervention targets by examining caregivers’ feelings of role captivity and grief. The third presentation will introduce the results of a pilot intervention , called “Building a Bridge,” which that improved caregivers’ knowledge and access to community resource. The final session will introduce a program based on the Acceptance and Commitment Therapy (ACT) model that reduces caregiver burden for community-dwelling parents and grandparents raising grandchildren or children in the community setting. Together, these presentations underscore the benefit of using strength-based models to relieve caregiver burden of caregivers. Savla , the discussant, will integrate key points from these interventions while and addressing considerations for future research to optimize outcomes for caregivers. This is a Mental Health Practice and Aging Interest Group Sponsored Symposium.

